# Hyperlactatemia of dialysis-dependent patients after cardiac surgery impacts on in-hospital mortality: a two-center retrospective study

**DOI:** 10.1186/s40981-020-00348-1

**Published:** 2020-06-11

**Authors:** Mariko Ezaka, Junko Tsukamoto, Koichi Matsuo, Nobuhide Kin, Kazue Yamaoka

**Affiliations:** 1 Department of Anesthesiology, New Tokyo Hospital, 1271 Wanagaya, Matsudo, Chiba, 270-2232 Japan; 2 Teikyo University Graduate School of Public Health, 2-11-1 Kaga Itabashi-ku, Tokyo, 173-8605 Japan; 3 Department of Anesthesiology, Saitama Red Cross Hospital, 1-5 Shintoshin, Chuo-ku, Saitama 330-8553 Japan; 4 Department of Intensive Care Unit, New Tokyo Hospital, 1271 Wanagaya, Matsudo, Chiba, 270-2232 Japan

**Keywords:** Dialysis-dependent, Cardiac surgery, Hyperlactatemia

## Abstract

**Background:**

Lactate is a well-known marker to estimate prognosis after cardiac surgery and critically ill patients. The liver and kidney have a major role in lactate metabolism; however, there was less characterized about the change of lactate and threshold to predict in-hospital mortality in dialysis-dependent patients undertaking cardiac surgery. We conducted this retrospective observational study to characterize when and how lactate values after cardiac surgery affected in-hospital mortality.

**Methods:**

This two-center retrospective study included dialysis-dependent patients who underwent cardiac surgery with a cardiopulmonary bypass from January 2014 to December 2018. Lactate values were collected at three points: at ICU admission (T1), the maximum level of lactate within 24 h postoperatively (T2), and 24 h after ICU admission (T3). We determined hyperlactatemia as more than 2 mmol/L following previous studies.

**Results:**

We enrolled 122 dialysis-dependent patients. The mean age was 73 ± 8 years and hyperlactatemia was observed in 100 patients (81.9%). In-hospital mortality was 11.4%. Univariate analysis and area under curve in ROC suggested that T2 lactate was the most significantly associated with in-hospital mortality (AUC = 0.845). Multivariate logistic analysis showed a significant association between in-hospital mortality when patients showed early peak lactate levels of > 4.5 mmol/L after ICU admission (adjusted OR 8.35; 95% CI: 1.44–57.13).

**Conclusions:**

In dialysis-dependent patients after cardiac surgery, the early-onset of a maximum arterial lactate concentration of > 4.5 mmol/L was significantly associated with in-hospital mortality.

## Introduction

The number of dialysis-dependent patients is increasing in Japan due to the increased longevity and prevalence of diabetes mellitus. In 2016, it reached > 300,000 patients with chronic dialysis, and > 39,000 new patients were registered yearly [1]. Along with aging, dialysis-dependent patients require cardiac surgery because calcification and continuous inflammation lead to valve stenosis and coronary artery disease, respectively [2]. Of 13,887 coronary artery bypass grafts (CABGs) in 2016 in Japan, 1608 (12%) were dialysis-dependent patients [3], and the number continues to rise. However, dialysis-dependent patients have higher mortality after cardiac surgery than non-dialysis patients [4–8]. The European system for cardiac operative risk evaluation II included dialysis as a risk factor for mortality [9]. They are more vulnerable to infection, pneumonia, abdominal ischemia, and infraction than non-dialysis patients [5, 7].

The kidney is the second most important organ after the liver for metabolizing and removing lactate from the circulation [10, 11]. It converts blood lactate to glucose in the Cori cycle and gluconeogenesis, and transformed glucose is utilized in tissue again [12]. If patients have chronic kidney disease, more hyperlactatemia and metabolic acidosis is anticipated. In previous studies, 20–80% of patients presented with hyperlactatemia after cardiac surgery, and increased lactate levels were associated with high mortality and long stays in the intensive care unit [13–15]. In those studies, the threshold of hyperlactatemia for in-hospital mortality was 3.0-4.4 mmol/L. A recent study showed that temporary hyperlactatemia less than 2.0 mmol/L within 24 h after cardiac surgery had an adverse impact on prognosis [16]; however, dialysis-dependent patients were few or excluded in these studies. We speculated that dialysis-dependent patients have higher blood lactate level due to lack of kidney function, and this high lactate level affects the mortality.

This two-center, retrospective, observational study including dialysis-dependent patients after cardiac surgery had two aims: to explore how the changes in arterial lactate levels are associated with in-hospital mortality and to find a threshold of lactate levels that predicts in-hospital mortality. Identifying the lactate trend and threshold help us deciding treatment strategies.

## Methods

### Setting and ethical approval

This two-center, retrospective, observational study was conducted at two middle-sized private hospitals (hospitals A and B). It was reviewed and approved by the ethics committee of New Tokyo Hospital (Matsudo, Chiba, Japan, approval ID: 0175) and that of the Saitama Red Cross Hospital (Omiya, Saitama, Japan, approval ID: 18- AO). This study was reported in accordance with the strengthening the reporting of observational studies in epidemiology statement [17].

### Patients

We enrolled dialysis-dependent patients who underwent elective open cardiac surgery from January 2014 to December 2018. The inclusion criteria were (1) age > 20 years, (2) chronically dependent on dialysis prior to cardiac surgery, (3) undergoing elective cardiac surgery with cardiopulmonary bypass (CPB). We excluded patients who required mechanical circulatory support, preoperative inotropes, and emergency surgery. Neither hospital performed cardiac transplantation or ventricular assisted device implantation. When patients underwent more than one cardiac surgery during the study period, we collected the data concerning the first operation.

### Intraoperative anesthesia and surgery

Anesthesia was administered according to the protocol of each hospital. Propofol or midazolam, and fentanyl were used for anesthesia induction; sevoflurane, fentanyl, and remifentanil were used for anesthesia maintenance. Rocuronium or vecuronium was used to achieve neuromuscular blockade. For all patients, arterial blood pressure was monitored using a radial or brachial artery catheter and central venous pressure was monitored using a central venous catheter. Transesophageal echocardiography was used to monitor the procedure and hemodynamic status. After heparinization (300 unit/kg), CPB was maintained under an activated coagulation time of > 400 s and at a cardiac index of 2.4-2.6 L/min/m. Intermittent cold cardioplegia was used for all patients. Hospital A used blood cardioplegia and hospital B primarily used crystalloid cardioplegia. Hemodialysis or extracorporeal ultrafiltration was combined with CPB to remove excess fluid. Mild systemic hypothermia (32–34 °C) was most often used; however, deep systemic hypothermia (25 °C in hospital A and 18 °C in hospital B) was used for circulatory arrest. The dose of inotropes and vasopressors to maintain hemodynamic stability depended on the attending anesthesiologist. Adrenaline, dopamine, dobutamine, noradrenaline, phenylephrine, and vasopressin were mainly used. All patients were intubated and admitted to the ICU along with mechanical ventilation support. After ICU admission, continuous hemodiafiltration (CHDF) or intermittent hemodiafiltration was performed depending on the patient’s hemodynamic and electrolyte status. We started intermittent hemodiafiltration in the morning on the day after the operation, whereas CHDF was implemented several hours after the operation, and the initiation time and duration of CHDF depended on each patient.

### Data collection

The main outcome of this study was in-hospital mortality of any causes. And blood lactate was measured from arterial blood gas samples (RAPIDLab 1200 Systems, Siemens Healthcare, Germany) after ICU admission. Blood gas samples were evaluated at least every 4 h until the patients were extubated or their hemodynamic status reached stability. In hospital A, the unit of arterial lactate measurement was mg/dl, thereby we divided the data by 9 to align mmol/L used in hospital B. We collected lactate measurements at three-time points: at ICU admission (T1), the maximum level of lactate within 24 h postoperatively (T2), and 24 h after ICU admission (T3). The time of T2 was also recorded.

The following data were collected as covariates for all patients from electronic medical records: age; sex; body mass index; preoperative complications including diabetes mellitus (DM), atrial fibrillation (Afib), cerebral ischemia (CI), coronary artery disease, and arteriosclerosis obliterans (ASO); year on dialysis; preoperative ejection fraction on transthoracic echocardiography; the cause of renal failure including DM, glomerulonephritis, nephrosclerosis; type of procedure including valve surgery, CABG, aortic surgery, complicated surgery with > 2 procedures; operating time; CPB time; aortic cross-clamp time; and use of adrenaline at ICU admission.

### Statistical analysis

Categorical and dichotomous variables were summarized as frequencies and percentages, respectively, and continuous variables as mean and standard deviation or median and interquartile range (IQR). To determine the most predictable arterial lactate levels for in-hospital mortality, we plotted receiver operating characteristic (ROC) curves and obtained the area under the curve (AUC). After deciding the most predictable arterial lactate levels from the AUC, univariate and multivariate logistic regression model with a stepwise variable selection method (inclusion and exclusion criteria: 0.2, respectively) were used for the analyses. We used log-transformed data of each lactate value because they did not follow a normal distribution. For the multivariate analysis, variables shown in Table 2 in addition to age and sex were used as covariates. We analyzed the association between arterial lactate and in-hospital mortality following 3 steps. First of all, we calculated the odds ratio in a multivariate logistic regression analysis using the continuous variable of lactate to explore whether the lactate is independently associated with in-hospital mortality. Secondly, we conducted additional multivariate logistic regression analyses to determine a threshold, each with lactate at different binary cutoff as a target value for intervention. Thirdly, we added the T2 time (< 12 h or > 12 h after ICU admission) on the second multivariate logistic regression analysis with the binary cutoff lactate level showing the highest odds ratio. All statistical analyses were performed using R Statistics version 3.3.0 (Institute for Statistics and Mathematics, Wirtschaftsuniversität Wien). A two-tailed *p* value < 0.05 was considered statistically significant.

## Results

### Patient characteristics

In hospital A, 120 dialysis-dependent patients underwent cardiac surgery during the study period. We excluded 7 patients who required emergency surgery and 10 patients because they did not require CPB for off-pump CABG. In hospital B, 36 dialysis-dependent patients were initially included and 17 patients were subsequently excluded (4 for emergency surgery and 13 for off-pump CABG). Therefore, we enrolled a total of 122 patients in this study. The patient characteristics are presented in Table 1. The patients had a mean age of 73 ± 8 years and a median year on dialysis of 9 (IQR 4–14) years. DM (35%) and nephrosclerosis (20%) were the primary reasons for dialysis.

**Table 1:** Preoperative patient characteristics

	Variates (*n* = 122)
Age (years)	73 (69,78)
Sex (male)	86 (71%)
BMI (kg/m^2^)	20.7 (18.5, 23.0)
Preoperative complications	
DM	50 (41%)
Afib	35 (29%)
CI	44 (36%)
CAD	31 (25%)
ASO	31 (25%)
Years on dialysis (years)	9 (4,14)
Ejection fraction (%)	49 (37, 63)
Cause of renal failure	
DM	43 (35%)
glomerulonephritis	25 (21%)
nephrosclerosis	13 (11%)
other	26 (21%)
unknown	15 (12%)

Data are presented as number (%), or median (1st quartile–3rd quartile)

*BMI* body mass index, *EF* ejection fraction, *DM* diabetes mellitus, *Afib* atrial fibrillation, *CAD* coronary artery disease, *CI* cerebral ischemia, *ASO* arteriosclerosis obliterans

**Fig. 1:**
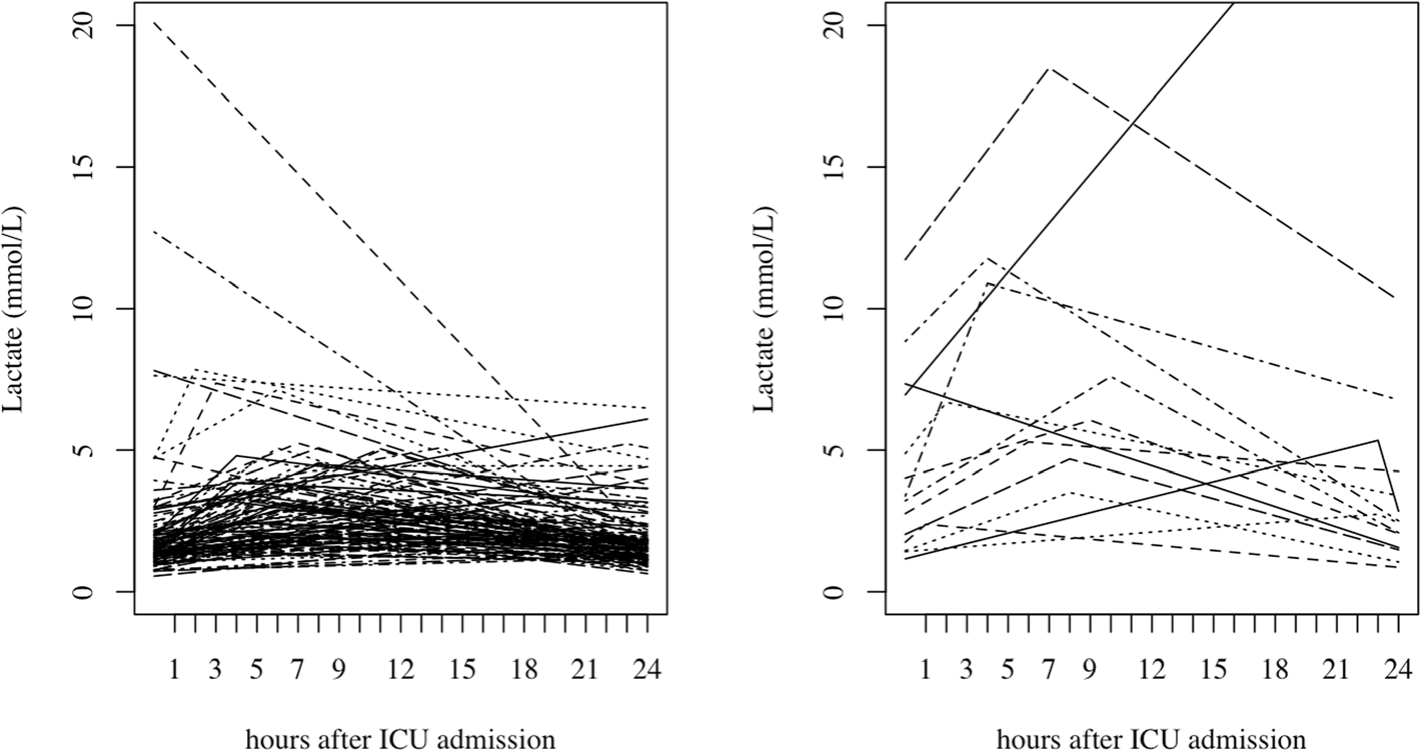
Arterial lactate levels within 24 h after ICU admission. Survivors (**a**) (*n* = 108) and non-survivors at hospital discharge (**b**) (*n* = 14)

In-hospital mortality and lactate levels, the in-hospital mortality was 11% (14 patients). Among those patients, the median hospital stay was 46 (IQR 21–78) days, and six out of fourteen patients (43%) died before the 30th postoperative day. Three patients with intestinal ischemia and one with cerebral ischemia were also included. Of all enrolled patients, 100 patients (82%) presented with hyperlactatemia (> 2 mmol/L) within 24 h postoperatively. The median arterial lactate levels at each time point were 1.57 mmol/L at T1, 3.12 mmol/L at T2, and 1.67 mmol/L at T3 (Table 2). The median time of T2 (the maximum lactate) was 8 h after ICU admission (Fig. 1).

**Table 2:** Univariate analysis of hospital mortality

	Survivor (*n* = 108)	Non-survivor (*n* = 14)	Odds ratio		
			Unadjusted	95% CI	*p* value
Lactate value					
T1 (mmol/L)	1.5 (1.2, 2.1)	3.3 (1.8, 6.4)	4.18	1.91-10.0	<0.001
T2 (mmol/L)	2.9 (2.0, 4.0)	6.4 (4.8, 10.0)	10.1	3.6-37.7	<0.001
T3 (mmol/L)	1.6 (1.2, 2.1)	2.4 (1.7, 4.0)	4.38	1.78-12.43	0.002
Peak time (hours)	8.0 (4.8, 14.0)	7.5 (4.0, 9.8)	0.98	0.91-1.06	0.75
Age	73 (69,77)	75 (69, 82)	1.05	0.98-1.15	0.18
Sex (male)	77 (71%)	9 (64.2%)	0.72	0.23-2.51	0.58
EF (%)	49 (37, 64)	56 (44, 60)	1.01	0.97-1.05	0.76
Years on dialysis	9 (3.5, 13.3)	13 (10, 17.8)	1.07	1.00-1.14	0.04
Preoperative complications					
DM	45 (42%)	5 (36%)	0.78	0.22-2.44	0.67
Afib	30 (28%)	5 (36%)	1.44	0.41-4.54	0.54
CI	35 (32%)	9 (65%)	3.75	1.20-13.0	0.03
CAD	27 (25%)	4 (29%)	1.20	0.30-3.92	0.77
ASO	25 (23%)	6 (43%)	2.49	0.75-7.85	0.12
Cause of renal failure					
DM	37 (33%)	6 (43%)	1.44	0.44-4.44	0.52
Procedure					
Valve	48 (45%)	5 (36%)	1.00		
CABG	11 (10%)	1 (7%)	0.87	0.04-6.17	0.91
TAR	3 (3%)	0 (0%)	0.00	NA	0.99
Complicated	31 (29%)	6 (43%)	1.85	0.51-6.95	0.34
Other	15 (14%)	2 (14%)	1.28	0.17-6.64	0.78
Operating time (min)	324 (241, 395)	468 (343, 519)	1.01	1.00-1.01	0.001
CBP time (min)	176 (140, 231)	242 (187, 354)	1.01	1.00-1.01	0.003
Clamp time (min)	130 (91, 172)	170 (139, 278)	1.01	1.00-1.02	0.003
Adrenaline (yes)	9 (8%)	5 (36%)	6.11	1.60-22.12	0.006
CHDF (yes)	86 (20%)	7 (50%)	3.9	1.22-12.60	0.02

Data are presented as number (%), or median (1st quartile–3rd quartile)

*T1* lactate at ICU admission, *T2* the maximum level of lactate within 24 h postoperatively, *T3* lactate at 24 h after ICU admission, *EF* ejection fraction, *DM* diabetes mellitus, *Afib* atrial fibrillation, *CAD* coronary artery disease, *CI* cerebral ischemia, *ASO* arteriosclerosis obliterans, *CABG* coronary artery bypass graft, *TAR* total arch replacement, *CPB* cardiopulmonary bypass, *CHDF* continuous hemodiafiltration

**Fig. 2:**
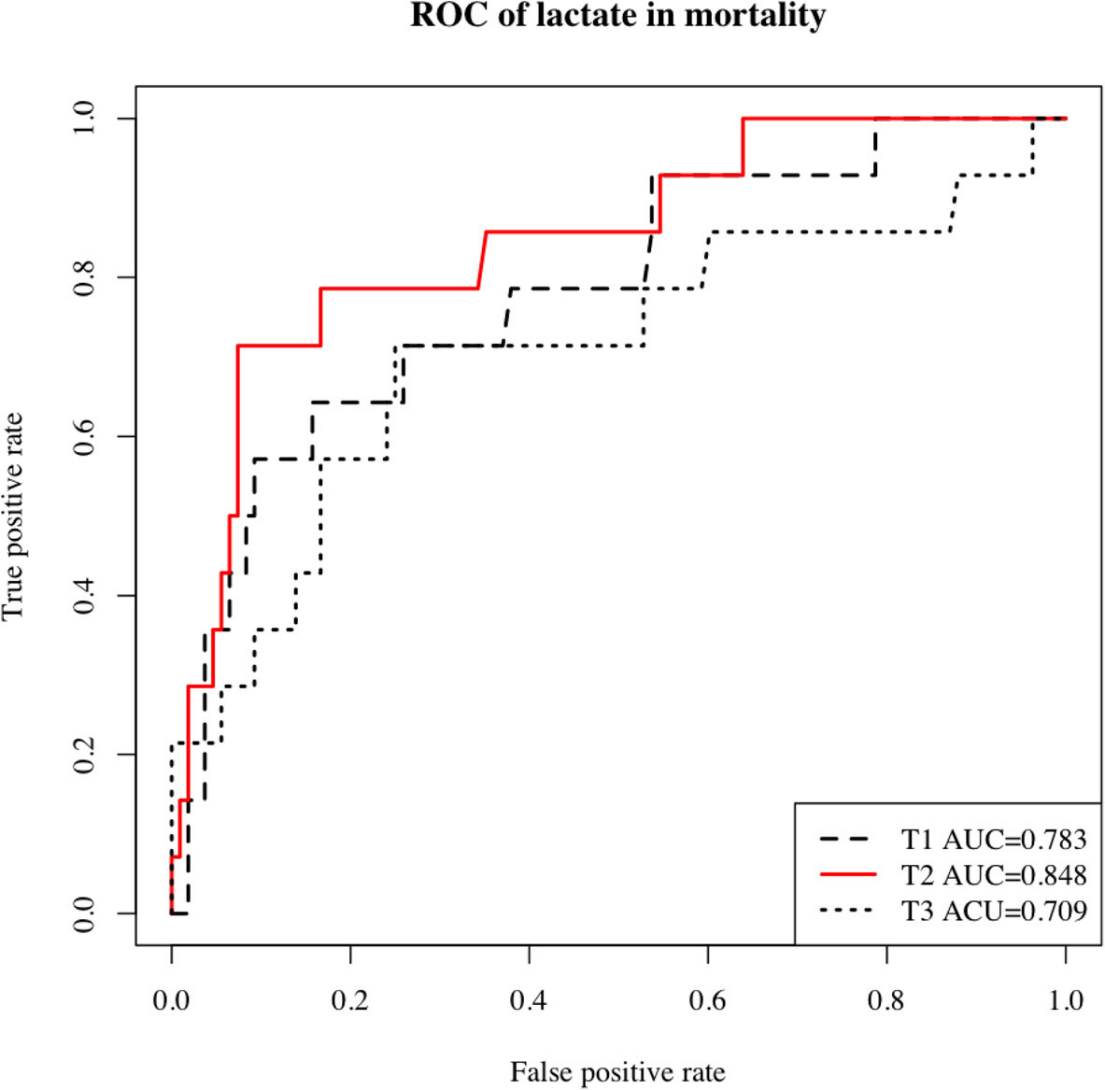
The area under receiver operating characteristics of lactate after ICU admission. *T1* lactate at ICU admission, *T2* the maximum level of lactate within 24 h postoperatively, *T3* lactate at 24 h after ICU admission

In the ROC analysis, T2 lactate levels showed the largest AUC (0.848, *p* < 0.001) (Fig. 2); therefore, we used the T2 lactate value in the logistic regression analysis. The univariate analysis of preoperative and intraoperative data revealed that age, preoperative history of CI and ASO, operating time, CPB time, aortic cross-clamp time, adrenaline use, and arterial lactate levels were associated with in-hospital mortality (Table 2). In multivariate logistic regression analyses, the T2 arterial lactate level was significantly associated with in-hospital mortality [odds ratio (OR), 6.01; 95% confidence interval (95% CI), 1.47–33.0] (Table 3). Table 4 displays additional multivariate logistic regression models at different binary thresholds. There appears to be a threshold effect at T2 lactate level of > 4.5 mmol/L. We also examined the association between in-hospital mortality and the combination of the T2 lactate (< 4.5 mmol/L or > 4.5 mmol/L) and the T2 time (< 12 h or > 12 h after ICU admission). The result indicated a significant association between in-hospital mortality when patients showed early peak lactate levels of > 4.5 mmol/L after ICU admission (adjusted OR 8.35; 95% CI: 1.44–57.13).

**Table 3:** Multivariable logistic regression analysis of hospital mortality (T2)

	Odds ratio	95% CI	*p* value
Model adjusted by age and sex	10.24	4.00-54.4	< 0.001
Model adjusted by age, sex, CI, CHDF, CPB time and years on dialysis	6.01	1.43-33.0	0.019

*CPB* cardiopulmonary bypass, *CI* preoperative history of cerebral ischemia, *CHDF* continuous hemodiafiltration

**Table 4:** Multivariable logistic regression analysis of hospital mortality (T2 binominal)

Variable of T2 lactate	Odds ratio	95% CI	*p* value
≥ 4 mmol/L	5.66	1.07-38.63	0.050
≥ 4.5 mmol/L	11.65	2.08-91.16	0.008
≥ 5 mmol/L	8.62	1.51-56.51	0.016
≥ 5.5 mmol/L	8.53	1.05-72.08	0.041

All models were adjusted by age, sex, CI, CHDF, CPB time, and years on dialysis. Models determined a threshold, each with lactate at different binary cutoff

## Discussion

### In-hospital mortality and lactate levels

This study was conducted to elucidate the changes and the threshold in arterial lactate levels associated with in-hospital mortality in dialysis-dependent patients. The main findings of our study indicated frequent hyperlactatemia in those patients and it was associated with in-hospital mortality when the maximum value was > 4.5 mmol/L within 12 h after cardiac surgery.

### Mechanisms of hyperlactatemia in dialysis patients

In general, lactate is a well-known marker that affects the prognosis after cardiac surgery. Although tissue hypoxia has been considered as the main causality of hyperlactatemia [18], accelerated glycolysis to create lactate to transform glucose also contributes to hyperlactatemia [4]. These two mechanisms of hyperlactatemia mean if a tissue needs more energy, a tissue itself and the whole metabolic system boost blood lactate level. While patients who died within 30th postoperative day had the highest lactate levels, we notice that late in-hospital deaths also had significantly higher lactate levels than survivors. This result indicates that hyperlactatemia within 24 h after cardiac surgery explains the comprehensive ability to manage tissue damage by hemodynamic change. Aside from these mechanisms, several factors should be considered for dialysis-dependent patients. Firstly, renal dialysis improves acidosis and controls hyperlactatemia in experimental situations [19], but its effect was controversial. In this study, although we used dialysis for all patients during CPB and after ICU admission, the blood lactate levels remained high in > 80% of patients. This suggests that hemodialysis was pragmatically inefficient in controlling overproduced lactate after cardiac surgery. Our result shows that CHDF after surgery was associated with higher mortality than intermittent hemodiafiltration because CHDF was prone to be chosen in patients with unstable hemodynamics. Efficacy of lactate removal between continuous or intermittent hemodiafiltration should be examined in future studies. Secondly, longer years on dialysis renders the arteries stiffer and leads to comorbidities such as peripheral arterial disease and DM [8], which may cause hypoxia-induced hyperlactatemia. This study did not reveal any association between peripheral arterial diseases such as ASO or DM, and in-hospital mortality. Although DM was a risk factor of perioperative mortality in non-dialysis patients, further investigation with a larger sample size is necessary to elucidate the causes of hyperlactatemia in dialysis-dependent patients. Lastly, dialysis-dependent patients are likely to have malnutrition because of strictly controlled diets [20]. Dialysis can induce vitamin deficiency, and the lack of vitamin B1 also induces hyperlactatemia [21]. Accelerated glycolysis in the absence of vitamin B1 causes lactate accumulation because pyruvate dehydrogenase cannot function. Considering those characteristics of dialysis-dependent patients, we should manage hyperlactatemia by diversified strategies. Recently, metabolic management [22] and microcirculation improvement [16] are examined for the effectiveness. Combination of thiamine, vitamin C, and corticosteroids was reported to improve lactate clearance and 28-day mortality in critically ill patients [22–24]. Luger et al. [25] showed patients without dialysis maintained their vitamin B1 level after cardiac surgery; however, there is no investigation in dialysis-dependent patients. Since the result of randomized clinical trial targeting thiamine intervention is controversial [26], further study is warranted to investigate the association between serum lactate levels and these interventions in dialysis-dependent patients.

### Strengths and limitations

This study showed that an arterial lactate level of 4.5 mmol/L was the threshold value above which in-hospital mortality increased linearly in dialysis-dependent patients. This is the first study that elucidates the dynamics of lactate levels in dialysis-dependent patients after cardiac surgery.

Our results also suggest that early-onset hyperlactatemia is associated with worse outcomes than late-onset hyperlactatemia and previous studies had controversial results [27, 28]. Early-onset hyperlactatemia was mainly associated with impairment of tissue oxygen during CPB [29], and late-onset was associated with mainly inotrope usage. Even though a cardiac index was maintained within 2.4-2.6 L/min/m2 in our study, longer CPB time and aortic cross-clamp time were associated with both in-hospital mortality and postoperative hyperlactatemia. Moreover, we used pulsatile flow that was proved to improve hyperlactatemia and reduce inotrope use intra- and post-operatively [30], our study suggests that it was not enough to diminish arterial lactate level in dialysis-dependent patients.

On the other hand, our study has several limitations to consider. Firstly, this was a retrospective study and we presented the association between hyperlactatemia and in-hospital mortality, but causation cannot be inferred. To prove this causation, intensive randomized controlled trial to reduce lactate levels is essential. Secondly, the sample size was limited; thus, we could not include several covariates in our multivariate logistic regression analysis. A larger multi-center study should be considered. We think this study is valuable that this result will give the important information about the effect size for the future RCT study. Thirdly, we could not obtain preoperative blood lactate levels in all enrolled patients. These levels were not measured in hospital A because of the lack of relevant equipment. To assume that preoperative lactate levels were within normal limits, we excluded emergency operations and patients who required inotropes preoperatively, whose preoperative lactate levels were high. By this, it could be expected to minimize the effects of preoperative variability of the lactate level.

## Conclusion

Among dialysis-dependent patients after cardiac surgery, the early-onset of a maximum arterial lactate concentration of > 4.5 mmol/L was significantly associated with in-hospital mortality.

## Data Availability

Derived data supporting the findings of this study are available from the corresponding author (ME) on request.
